# Maximizing Utility or Avoiding Losses? Uncovering Decision Rule-Heterogeneity in Sociological Research with an Application to Neighbourhood Choice

**DOI:** 10.1177/00491241231186657

**Published:** 2023-07-18

**Authors:** Ulf Liebe, Sander van Cranenburgh, Caspar Chorus

**Affiliations:** 1 Department of Sociology, 164352 University of Warwick, Coventry, UK; 2 Faculty of Industrial Design Engineering (IDE), and Faculty of Technology, Policy and Management (TPM), Delft University of Technology, Delft, The Netherlands

**Keywords:** decision rules, loss aversion, rational choice, regret minimization, utility maximization, stated choice experiment

## Abstract

Empirical studies on individual behaviour often, implicitly or explicitly, assume a single type of decision rule. Other studies do not specify behavioural assumptions at all. We advance sociological research by introducing (random) regret minimization, which is related to loss aversion, into the sociological literature and by testing it against (random) utility maximization, which is the most prominent decision rule in sociological research on individual behaviour. With an application to neighbourhood choice, in a sample of four European cities, we combine stated choice experiment data and discrete choice modelling techniques and find a considerable degree of decision rule-heterogeneity, with a strong prevalence of regret minimization and hence loss aversion. We also provide indicative evidence that decision rules can affect expected neighbourhood demand at the macro level. Our approach allows identifying heterogeneity in decision rules, that is, the degree of regret/loss aversion, at the level of choice attributes such as the share of foreigners when comparing neighbourhoods, and can improve sociological practice related to linking theories and social research on decision-making.

## Introduction

While early sociology was interested in choice behaviour as an important part of sociological analysis (see Hinkle 1963), this interest seemed to have decreased considerably over time. Not many sociologists nowadays consider developments in decision-making research and choice modelling. As Bruch and Feinberg (2017) mention this might be due to the fact that sociological explananda differ from the explananda in decision-making research and that the latter typically ignores social contexts when studying decision-making. Yet, choice modelling research – the modeling of decision processes and outcomes – can help to uncover how individual behaviour varies across social contexts as well as the microfoundations of many macrophenomena. One of the most striking examples of this is residential segregation (Sakoda 1971; Schelling 1971), where subtle behavioural responses at a microlevel are well known to have the potential to lead to severe consequences such as ethnic segregation at the macrolevel. A good understanding of individual decision-making is therefore a central part of micro as well as micro–macro explanations in sociology and other social sciences (Coleman 1990; Raub et al. 2011; Schelling 1978; Wippler and Lindenberg 1987).

Theoretical (formal) models of individual decision-making include specific behavioural assumptions to describe choice behaviour, and more generally, some sort of behavioural choice theory underlies much of sociological work on human action. The most common assumption across the social sciences, including rational choice theory in sociology (Hechter and Kanazawa 1997; Kroneberg and Kalter 2012; Voss and Abraham 2000), is linear–additive utility maximization. It is this specification that has also gained immense popularity in empirical, microeconometric analyses of choice behaviour, as facilitated by the emergence of discrete choice theory and its dominant branch of random utility theory (RUT) and its dominant linear-in-parameters specification of utility functions (Ben-Akiva and Lerman 1985; McFadden 1973; Train 2009). This theory postulates that, when faced with different choice alternatives the actor chooses the alternative that yields the greatest utility. Aside from a random utility component that captures aspects of decision-making outside the model's realm, an alternative's utility is formalized as a weighted sum of the alternatives’ attributes and their associated weights. Such discrete choice models – modelling the choice among a set of alternatives – have been applied in social science research (see Liebe and Meyerhoff 2021 for an overview), for example, to examine: how offenders choose target areas (Bernasco and Block 2009); employment outcomes on labour markets (Logan 1996); voting turnout (Zeng 2000); postmaterialist value priorities (Moors and Vermunt 2007); intergroup friendship choice (Zeng and Xie 2008); residential mobility and locational attainment (Quillian 2015); and discrimination (Liebe and Beyer 2021).

While rational choice theory in general and RUT with linear-in-parameter utility functions in particular can have high explanatory power and provide a close link between theory and quantitative methods (Bruch and Feinberg 2017: 2010), they are often criticized for being too simplistic and unrealistic from a behavioural viewpoint (e.g., Chorus 2014; Coleman and Fararo 1992; Hensher et al. 2018; Kahneman and Tversky 2000). This means behaviour might often be better described by alternative decision rules, but this is not well reflected in sociological research because researchers typically do not consider behavioural assumptions at all or (implicitly) assume a RUT specification. Recent works in discrete choice theory have pioneered the systematic examination of decision rules and mixtures of different decision rules in a population (Hensher et al. 2018; Hess and Chorus 2015; Hess et al. 2012; van Cranenburgh and Alwosheel 2019; Zhu and Timmermans 2010); these models have the potential to enrich sociological research by uncovering the heterogeneity of decision-making processes in a population.

In this article, we introduce one of these alternative decision rules, namely random regret minimization (RRM), in the sociological literature and demonstrate how different decision rules – utility maximization and regret minimization – can be empirically inferred from observed choices, by combining choice modelling with stated choice experiments (SCEs). With neighbourhood choice as an example, we examine the relative prevalence of these two decision rules, the stability (or variation) of decision rule prevalence across social/city contexts, and exemplify the differences in neighbourhood demand at the macrolevel that may result from different decision rules. This illustrates that (assumptions about) decision rules potentially matter for societal outcomes.

RRM is closely related to loss aversion (Tversky and Kahneman 1991), which is a fundamental alternative to random utility maximization (RUM) and has been shown to be a universal phenomenon (Mrkva et al. 2019). For example, regarding crime and deviance, Thomas and Nguyen (2022) demonstrate, based on a survey of college students in the United States, that participants were more willing to engage in (hypothetical) deviant behaviour (fighting, drunk driving and marijuana use) to prevent the loss of social status compared with gaining social status. For the case of educational decisions, with implications for educational inequalities, Barone et al. (2021) show that loss aversion is a more realistic behavioural foundation than the downward mobility assumptions in the Breen–Goldthorpe model. This is empirically supported in a survey of school students from Paris, France. In a series of laboratory experiments, Molm (1997) demonstrates the importance of loss aversion for the use of coercive power in social exchange relations.

RRM and Tversky and Kahneman's (1991) model of loss aversion postulate that reference points matter for decision-making and that losses with respect to that reference point loom larger than gains of equal size. Instead of maximizing utility, individuals are assumed to minimize anticipated regret when comparing choice alternatives such as different neighbourhoods regarding access to public transport, environmental quality and ethnic composition.

In terms of the collection of data to estimate and validate models of choice behaviour, SCE techniques have been developed that accommodate the intuition that, when facing decisions, individuals typically have to consider different relevant factors or attributes of choice alternatives (Auspurg and Hinz 2015; Hensher et al. 2005; Liebe et al. 2021; Louviere et al. 2000). Take for example residential location decisions, which involve trade-offs between attributes such as noise pollution and distance to the city centre on the housing market, or income and work and commute time at the labour market. SCEs – in which individuals are asked to choose between options that vary in their attribute levels – can be used empirically to shed more light on the relative importance of attributes to decision-makers as well as the trade-offs they make between attributes. These are key advantages over using simple survey items to measure the importance of theoretically relevant factors in a decision-making context (Auspurg and Hinz 2015; Liebe and Meyerhoff 2021). Furthermore, the experimental control over the attribute variation in the experiment is an advantage over revealed preference data (e.g., market data); however, the latter is preferable in terms of external validity. The combination of discrete choice theory and its variants, SCEs and choice modelling provides a close link between theory, preference measurement and statistical model (Liebe et al. 2021; Louviere et al. 2010). This will be shown in the following for two competing decision rules with neighbourhood choice as study context.

### Neighbourhood Choice as Study Context

Our context of study is neighbourhood choice, a decision context that is known to include complex decision-making processes. Influenced by Schelling's (1971) work on segregation, neighbourhood preferences and choice have been studied using various approaches (see Bruch and Mare 2012) including survey data (e.g., Krysan and Bader 2007; Quillian 1995), vignette studies (e.g., Emerson et al. 2001; Howell and Emerson 2018), agent-based models (e.g., Bruch and Mare 2006; Fossett 2006) and SCEs (Ibraimovic and Hess 2017; Ibraimovic and Masiero 2014). While patterns of segregation at the aggregate level are often the main explanandum, understanding individual preferences and decision-making is an integral part of this research. In other words, knowing the decision-making processes at the microlevel is important to understand how the observed macrolevel outcomes are generated. For example, Bruch and Mare (2006) employ an agent-based model to investigate the dynamics of residential segregation and test several assumptions about ‘how individuals evaluate their neighbourhood’ (p. 682). They use vignette data or stated preference data, respectively, to find out which assumptions provide the best picture of how individuals make their actual choices. They find that continuous functions are empirically more plausible than (Schelling's) threshold functions.

Ibraimovic and Hess (2017) examine individuals’ preferences for neighbourhoods in Lugano, Switzerland, using an SCE (see also Ibraimovic and Masiero 2014). In this experiment, participants chose repeatedly between three hypothetical neighbourhood alternatives the one they prefer most. These alternatives varied, among others, in the concentration of conationals and share of foreigners. Based on latent class choice models, Ibraimovic and Hess (2017) found that the sample can be separated into three classes differing in their preferences. There exist two classes with a positive preference for conationals and one class, which does not significantly value conationals. The concentration of foreigners has an impact in only one class. Swiss citizens in this study disvalue an increase in foreigner share more than non-Swiss citizens. In this work, the authors assume a rational decision-maker, who maximizes utility through a linear weighting of relevant attributes (Ibraimovic and Hess 2017: 10).

Bruch and Swait (2019) analyse residential mobility and racial segregation by developing a choice set formation (CSF) model. Contrary to standard models in which individuals consider all alternatives and attributes, the CSF model assumes a multiple stages process in which boundedly rational actors first select a reduced set of choice alternatives and then select the alternative they prefer most. Based on L.A.FANS panel data of households living in 64 sampled neighbourhoods in Los Angeles County, that is, revealed neighbourhood preferences data, they find that the CFS models fit the residential mobility data better than a standard conditional logit model. Choice sets – neighbourhood alternatives considered by individuals – differ between racial and income groups. Blacks and Hispanics and low-income groups are more likely to consider neighbourhoods with a disproportionate share of their own groups. Using an agent-based model, Bruch and Swait (2019) further demonstrate that racially stratified choice sets can boost racial segregation.

Similar to previous research, in our study we cannot consider all the complexity of neighbourhood choice processes, including multistage decision-making. We work with a rather simple but still realistic example to demonstrate the merits of choice modelling and SCE research in sociology, with a focus on examining decision rules and decision rule-heterogeneity in decision-making. In our study, we pay attention to neighbourhood attributes such as environmental quality and ethnic composition (foreigner share), where the latter is related to segregation. In this regard, employing a SCE has the advantages that the importance of foreigner share is not analysed in isolation and that individuals are asked to make trade-offs when deciding between neighbourhood alternatives. Together with related research on social contexts and decision-making (Bruch and Feinberg 2017), this can be seen as a starting point of a research agenda bridging sociology and choice modelling.

In the following, we discuss two decision rules, decision rule-heterogeneity as well as an approach to model such heterogeneity. Then we describe our SCE on neighbourhood choice, the corresponding data and variables, and present the results. The article concludes with a summary of our findings and a discussion for future research.

## RUM, RRM and Decision-Rule Heterogeneity

Although the dominance of the linear additive utility maximization paradigm is undisputed in the field of discrete choice theory, alternatives have been proposed to capture well-known deviations from rationality. For example, the existence of so-called compromise effects and other decoy effects (Simonson 1989), which are well documented in consumer choice contexts, has inspired several extensions and alternatives to the dominant utility-maximization paradigm (e.g., Chorus and Bierlaire 2013; Guevara and Fukushi 2016; Kivetz et al. 2004; Leong and Hensher 2015). A great variety of alternative decision rules – either framed as extensions or adaptions of the standard model or based on entirely different theories – have been and continue to be developed (see Chorus 2014 and Hensher et al. 2018 for overviews). In this article, we focus on one alternative to RUM and its mainstream linear additive utility maximization specification: RRM (Chorus 2010; van Cranenburgh et al. 2015). RRM has been selected for its strong ties to behavioural theories, intuitive interpretation in the context of neighbourhood choice as well as its pragmatic and tractable model specification. In the remainder of this section, we will present the RUM and RRM models of choice behaviour that will be used for our empirical analysis, followed by a formal account of the Latent Class framework, which we use to model heterogeneity in decision rules.

### Linear-in-Parameters RUM

The linear additive, or linear in parameters RUM model (also the standard model) assumes that the decision-maker assigns a utility to each alternative, which is based on a weighted summation of attributes of alternatives; the weights are estimable parameters that represent the marginal utility, that is, the change in utility caused by a unit change of the attribute level. Crucially, this model thereby assumes that decision-making is fully compensatory, in that a deterioration of one attribute can always be compensated by an equally large improvement of another attribute that is equally important, or for example, by a twice as large improvement of another attribute that is half as important.

Since the analyst does not observe everything that matters to the decision-maker upon making a choice, the analyst cannot perfectly predict the utility assigned by a particular individual to a particular alternative. To capture this so-called unobserved part of utility, a (stochastic) error term is added to the model. As such, total utility becomes the sum of the systematic (linear additive) part and the error term, and choice is then defined up to a probability. Depending on the specified distribution for this random error term, different formulations for choice probabilities are obtained. Under the assumption that *ε* is iid Extreme Value Type I, the widely used logit is obtained:

$$U_{in}=V_{in}+\varepsilon_{in}=\underset{m}{\sum }\;\beta_{m}\cdot x_{imn}+\varepsilon_{in}$$

$U_{in}$ denotes the total utility associated with considered alternative *i* by decision-maker *n*. $V_{in}$ denotes the systematic utility associated with *i* by decision-maker *n.* $\varepsilon_{in}$ denote the error terms associated with *i* by decision-maker *n*; if it is assumed that it follows an i.i.d. Extreme Value Type I distribution, practical logit type choice probabilities ( P ) are obtained, assuming *J* alternatives in the choice set: $P_{in}=\exp(V_{in})/\sum_{\;j=1..J}\;\exp(V_{\;jn})$ . $\beta_{m}$ denotes the taste parameter (decision weight) associated with attribute $x_{m}$ . $x_{imn}$ denote the values associated with attribute *m* for the considered alternative *i* by decision-maker *n*.

For example, Bernasco and Block (2009: 114) explicitly linked discrete choice models (conditional logit) and hence RUT to a rational actor framework with a utility equation consisting of several theoretical factors to study crime location choices: ‘[a]ssuming that robbers choose the tract they favour most after taking into account the pros and cons […]’. Other examples of explicit links between RUT choice models (here conditional logit model) and rational choice theories in sociological research include Xie and Shauman's (1997) study on sex-typing of occupational choice, which among others explicitly refers to an expectation model, and Stocké's (2007) study testing predictions of the Breen–Goldthorpe model of educational attainment.

### RRM and Loss Aversion

While loss aversion and prospect theory (Tversky and Kahneman 1991) are acknowledged as an important behavioural phenomenon and theory across the social sciences, there are fewer empirical applications, compared with rational choice models referring to utility maximization, in sociology, political science and other social sciences. One reason could be that researchers tend to ignore behavioural assumptions when investigating the factors of decision-making. This was for example highlighted by Mercer (2005: 1) in the context of political science research: ‘it suggests that prospect theory's failure to ignite the imagination of more political scientists probably results from their aversion to behavioural assumptions and not from problems unique to prospect theory.’ Although loss aversion and corresponding theories have been questioned in decision-making research in the last decades (e.g., Gal and Rucker 2018), there is robust evidence that people tend to be loss averse (e.g., Mrkva et al. 2019), and loss aversion is also discussed in the context of internal migration and housing decisions (e.g., Clark and Lisowski 2017; Morrison and Clark 2016).

The RRM model (Chorus 2010; van Cranenburgh et al. 2015) is related to the phenomenon of loss aversion (albeit different from prospect theory) and assumes that attributes of competing alternatives serve as reference points which are used to evaluate a considered alternative: more specifically, the model postulates that choices are determined by the wish to minimize anticipated regret – regret being conceptualized as the emotion that is felt when one or more non-chosen alternatives perform better than the chosen one, in terms of one or more attributes. The regret associated with an alternative equals the sum of all so-called binary regrets that are associated with bilaterally comparing the considered alternative with each of the other alternatives in the choice set. The level of binary regret associated with comparing the considered alternative with another alternative is taken to be the sum of the regrets that are associated with comparing the two alternatives in terms of each of their attributes. This attribute-level regret in turn is either (close to) zero when the other alternative performs worse than the considered alternative in terms of the attribute, and it grows as an approximately linear function of the difference in attribute values in case the considered alternative performs worse than the alternative with which it is compared. In that case, an estimable parameter (for which also the sign is estimated) gives the approximation of the slope of the regret function for the attribute. In formal notation, in its most generic form, the total regret of a considered alternative is written as follows:

$$RR_{i}=R_{i}+\upsilon_{i}=\underset{\;j\neq i}{\sum }\;\underset{m}{\sum }\;\mu \cdot \left[\ln\;\left(1+\exp\left[\frac{\beta_{m}}{\mu }\cdot (x_{\;jm}-x_{im})\right]\right)\right]+\upsilon_{i}$$

**Figure 1.**
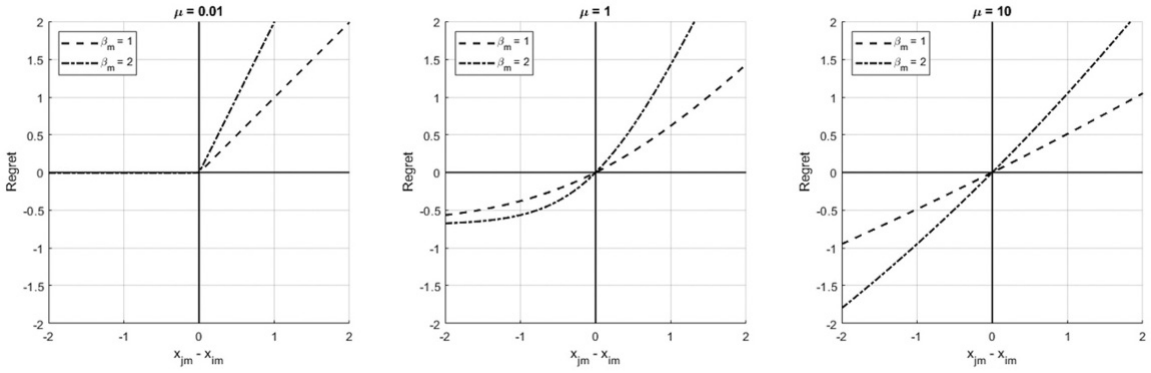
Effect of the regret aversion parameter µ on the attribute-level regret function.

$RR_{i}$ denotes the total regret associated with considered alternative *i*. $R_{i}$ denotes the systematic regret associated with *i*. $\upsilon_{i}$ denotes the error terms associated with *i*; if it is assumed that its negative follows an i.i.d. Extreme Value Type I distribution, practical logit type choice probabilities ( P ) are obtained, assuming *J* alternatives in the choice set: $P(i)=\exp(-R_{i})/\sum_{\;j=1..J}\;\exp(-R_{j})$ . $\beta_{m}$ denotes the taste parameter (decision weight) associated with attribute $x_{m}$ . $x_{im},x_{\;jm}$ denote the values associated with attribute *m* for, respectively, the considered alternative *i* and another alternative j . μ is a regret aversion parameter. This parameter governs the shape of the attribute-level regret function, as shown in Figure 1 below. If μ approaches 0, only regret matters and its behavioural counterpart, ‘rejoice’ is irrelevant (left-hand side plot). If μ becomes (very) large, regret and rejoice are equally important (right-hand side plot). In that case, the RRM model is indistinguishable from a linear-in-parameter multi-attribute utility maximization model. A regret aversion parameter of size 1 implies moderate regret aversion as in the conventional RRM model (middle plot) (Chorus 2010). Note that to ease comparison, the functions are shifted along the *y*-axis such that *R* = 0 for *x_jm_* – *x_im_* = 0 (see Chorus and Van Cranenburgh 2018 for a discussion on this topic).

The linear in parameters RUM and the RRM model substantially differ in the assumed decision-making process. In the linear in parameters RUM model, the decision-maker is assumed to ‘compute’ the utility of the choice alternatives by means of combining/multiplying their tastes/decision-weights with alternative-specific attribute-values. For example, their taste for distance to public transportation is combined with the distance to public transportation of a neighbourhood choice alternative and the same is done for other neighbourhood choice attributes such as the share of foreigners. The sum of these combinations forms a measure of utility of a neighbourhood choice alternative. After having repeated this process and computed utilities for all neighbourhood choice alternatives, the utilities are compared and the neighbourhood choice alternative with the highest utility is chosen.

The RRM model assumes a different decision-making process. For example, in the context of neighbourhood choice all neighbourhood choice alternatives are bilaterally compared on the choice attributes distance to public transportation, foreigner share, etc. The decision-maker is assumed to combine their taste/decision-weights for distance to public transportation with differences in distance to public transportation in each bilateral comparison of neighbourhood choice alternatives (first neighbourhood vs. second neighbourhood, first neighbourhood vs. third neighbourhood, etc.). This process is done both ways (first neighbourhood vs. second neighbourhood and second neighbourhood vs. first neighbourhood, etc.). After having repeated this process for all choice attributes, the sum of all choice attribute-level regrets for each neighbourhood choice alternative can be computed. These measures of overall regret per neighbourhood choice alternative are compared, and the alternative with the minimum regret is chosen.

The attribute-level regret aversion parameter μ provides more insights into the extent of differences between the regret, for example, from a loss in (short) distance to public transportation and the rejoice from an equivalent gain in (short) distance to public transportation. If there are hardly any or no differences (i.e., linear regret function; Figure 1, right-hand side), RRM and RUM result in the same neighbourhood choice probabilities (although based on different assumptions on decision processes). If the differences are very large, this would lead to pure random regret (Figure 1, left-hand side), and moderate differences would imply a regret function between the two ‘extremes’ linear regret and pure random regret. For example, if decision-makers have a dislike for a high share of foreigners in the neighbourhood, moderate regret means when a neighbourhood choice alternative has a high share of foreigners, while a competing neighbourhood choice alternative has a low share of foreigners, this causes a considerably amount of regret, while a considered neighbourhood choice alternative with a low share of foreigners and a competing neighbourhood choice alternative with a high share of foreigners causes a low amount of rejoice. Therefore, depending on the attribute-level regret aversion parameter and hence attribute-level regret function, decision-making regarding a neighbourhood choice attribute might be not at all, moderately, or completely in line with RRM.

Another key difference between the random regret model and its utility-based counterpart is that the regret model is semi-compensatory: the extent to which an attribute's deterioration can be compensated by an improvement in another attribute, depends on the relative position of the alternative in the choice set, in terms of the attributes. When an alternative already performs poorly on an attribute compared to other alternatives, a further deterioration in the attribute generates a lot of regret and cannot be easily compensated by an improvement of another equally important attribute, especially when the alternative already performs well on the improved attribute. This type of semicompensatory behaviour finds its roots in the convex regret function and has been found to generate a range of empirically well-established findings such as a preference for so-called compromise alternatives which have an intermediate performance on most attributes (Chorus and Bierlaire 2013). As is explained in more detail in Chorus and van Cranenburgh (2018), it is instructive at this point to observe the relation between the behaviour implied by the RRM model, and the notion of loss aversion in riskless (1991). The two models both postulate that reference points matter for decision-making and that losses with respect to that reference point loom larger than gains of equal size. A crucial difference between the two models is the choice of reference point: whereas the loss aversion model uses the status quo as a reference point, the RRM model uses the attributes of other alternatives in the choice set as reference points.

### Decision Rule-Heterogeneity

Our investigation into behavioural heterogeneity is structured as follows: we provide a detailed empirical exploration of the standard model. Then, we explore how well the regret minimization model in isolation predicts neighbourhood choices, compared to the standard model. In addition to this comparison, we then combine the RUM and RRM models into a latent class (LC) model, which postulates that choices made by different individuals may be represented by different decision models or rules (utility maximization or regret minimization). Each individual in the sample – and the choices they make – then belong to either a utility maximization class or a regret minimization class. The resulting latent class model estimates the probability of a randomly sampled individual belonging to either class, as well as – for each class – a set of taste parameters (decision weights $\beta_{m}$ ) of relevant attributes. It has to be stressed that, because decision rules and parameter sets vary across classes, heterogeneity in decision rules becomes empirically confounded with taste heterogeneity. In the following section, we will further elaborate on this. In notation, our two-class (aka discrete mixture) model may be described as follows:

$$P(i)=\pi_{r}P(i|r)+\pi_{u}P(i|u)$$

That is, the probability that a randomly sampled individual chooses alternative *i* equals the weighted sum of the probabilities that they would choose the alternative if they would belong to the utility ( u ) class and the regret ( r ) class, respectively; these choice probabilities are given by $\exp(V_{i})/\sum_{\;j=1..J}\;\exp(V_{j})$ for the utility class, and by $\exp(-R_{i})/\sum_{\;j=1..J}\;\exp(-R_{j})$ for the regret class, where *V* and *R* is defined as in equations (1) and (2), respectively. The weights π reflect the probability of belonging to either class and are estimated indirectly by means of a binary logit function; $\pi_{r}=\exp(\delta_{r})/\exp\;(\delta_{r})+1$ , and $\pi_{u}=1-\pi_{r}$ .

## SCE on Neighbourhood Choice

**Figure 2.**
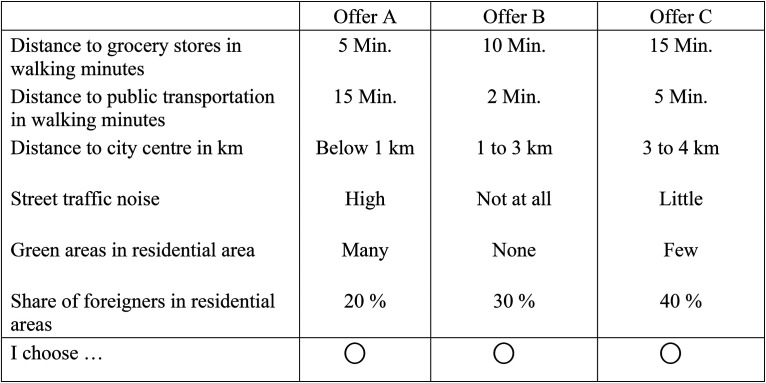
Example of a choice-set in the choice-experiment.

In the SCE, we asked the respondents to assume that they are moving to another city and search for a flat/house. They were further asked to assume that they have found three offers which are similar regarding size of the flat, facilities, and price. Yet the residential neighbourhoods, in which the flats are located, differ in several attributes. These attributes include distance to grocery stores, public transport, and the city centre in walking minutes; as well as noise exposure, share of green areas, and share of foreigners in percent (see Table 1). All attributes were described by four levels. Each choice set contained three alternatives which varied in the attribute levels (see Figure 2 for an example). To force trade-offs between the choice attributes, we employed an optimal orthogonal in the differences design taking all main and two-way interaction effects into account. This resulted in 32 choice sets (a design d-optimality of 100%). Each of the 2,430 respondents answered four randomly chosen choice sets, resulting in 9,720 choice observations. A balance check indicates that randomization worked and the exposure to choice sets and hence choice attributes is independent from respondents’ characteristics such as gender, age, education, income and migration background (see Table A1 in the Online Appendix, the highest correlation amounts to |0.008|).

**Table 1.** Attributes and Levels in the Choice Experiment.

Attribute	Levels	Variable
Distance to grocery stores in walking minutes	2 min., 5 min., 10 min., 15 min.	Stores
Distance to public transportation in walking minutes	2 min., 5 min., 10 min., 15 min.	Transport
Distance to city centre in km	Below 1 km, 1 to 2 km, 3 to 4 km, over 4 km	City
Street traffic noise	None, little, medium, high	Noise
Green areas in residential area	None, (very) few, some, many	Green
Share of foreigners in residential areas	10%, 20%, 30%, 40%	Foreigner

### Data and Variables

The SCE was part of an online survey that was conducted from November 2017 to January 2018 in the cities of Hannover (Germany), Mainz (Germany), Bern (Switzerland), and Zurich (Switzerland).^1^ This online survey was implemented as a follow-up survey to a study carried out one year earlier. The original survey was designed as a German-language mail survey. Both the original and the follow-up survey were announced as surveys on ‘Housing and Living in (City X)’. Neither the topic of environmental justice nor the parallel survey in the other city were mentioned at any time. The questionnaires and invitation letters were only slightly adjusted to the respective study area in terms of sender, date, picture on the cover, city name and language use. The latter was necessary to accommodate slight language differences between Germany and Switzerland.

For the original mail survey, random samples of 4,000 residents per city were drawn from the cities’ population registers. The samples included foreigners and were restricted to those aged between 18 and 70 years who are neither homeless nor living in collective households such as residential homes or prisons. The resulting response rates were 34.9% for Hannover (*n* = 1,604), 45.2% for Mainz (*n* = 1,800), 55.2% for Bern (*n* = 2,196) and 48.4% for Zurich (*n* = 1,931), respectively (standard RR2 for mail surveys to specifically named persons, AAPOR, 2016). While the study was not promoted as a panel, the mail questionnaire explained that a follow-up study was planned and that respondents could indicate if they did not want to participate any further. A year later, those who had not opted out were sent a postal invitation to the follow-up online survey (*n* = 1,106 in Hannover; *n* = 1,329 in Mainz; *n* = 1,628 in Bern; *n* = 1,359 in Zurich). When needed, up to two reminders were used. This resulted in response rates of 58.2% for Hannover (*n* = 641), 45.6% for Mainz (*n* = 605), 51.3% for Bern (*n* = 830) and 49.3% for Zurich (*n* = 668). For the purposes of this analysis, the sample was further narrowed down to those without missing values regarding the choice experiment. Furthermore, all those who indicated they had moved since the first survey were removed. The resulting total sample size for the choice experiment across cities amounts to 2,430 (*n* = 431 for Hanover; *n* = 573 for Mainz; *n* = 790 for Bern; *n* = 636 for Zurich). The sample is not representative for the corresponding cities. Yet, our main purpose is the analysis of heterogeneity in decision rules based on an SCE, which does not necessarily imply representative data (Mutz 2011). About half of our respondents (54%) are women, mean age amounts to 45 years (mean = 45.32, *SD* = 13.47, min/max = 18/72), and on average respondents have 16 years of education (mean = 15.72, *SD* = 2.36, min/max = 8/18), which indicate a high share of respondents with higher education. The average income (net monthly equivalent household income per capita in Euro, PPP adjusted) is 2,980.60, *SD* = 1,449.00, min/max = 286.70/8,600.92 (*n* = 2,076). The proportion of respondents with a Western/European migration background is 9.95%, and the one with a non-Western background (Africa/Asia/South America) is 15.89.

## Results

### Linear-in-Parameters RUM

Table 2 reports the results of the standard model assuming linear in parameters utility maximization. The underlying statistical model is a conditional logit model including the choice attributes distance to grocery stores, public transport and the city centre; as well as noise exposure, share of green areas and share of foreigners. Note that given the purpose of our study, we treat all attributes in our data as interval variables. We are aware that – technically speaking – the variables city, noise and green are ordinal in nature. However, RRM models only diverge from its utility counterpart in case of attributes that are interval in nature. Therefore, treating them as ordinal variables would undermine the purpose of our analysis (see Section 4.1.3 in Chorus 2012 for an extended discussion of the treatment of noncontinuous [categorical] variables in RRMs). Table 2 (RUM model) shows all attributes, except the share of green areas, have a negative effect on the probability of choosing a neighbourhood. This means a neighbourhood is less attractive if it is further away from grocery stores, public transport and the city centre, and if the noise exposure and the share of foreigners are higher. On the other hand, a neighbourhood is, ceteris paribus, more attractive if the share of green areas is higher.

**Table 2.** Random Utility Maximization (RUM) and Random Regret Minimization (RRM) Models.

	RUM	muRRM	muRRM
*Choice attributes*			
Stores	−0.0344*** (0.003)	−0.0258*** (0.002)	−0.0281*** (0.002)
Transport	−0.0740*** (0.003)	−0.0530*** (0.002)	−0.0534*** (0.002)
City	−0.167*** (0.009)	−0.128*** (0.006)	−0.131*** (0.006)
Noise	−0.437*** (0.012)	−0.347*** (0.008)	−0.349*** (0.009)
Green	0.416*** (0.012)	0.284*** (0.008)	0.285*** (0.008)
Foreign	−1.196*** (0.011)	−1.010*** (0.076)	−0.990*** (0.076)
*Regret aversion parameters*			
μ		0.158*** (0.017)	
μ stores			10 (fixed)
μ transport			0.187*** (0.056)
μ city			0.243*** (0.078)
μ noise			0.05 (fixed)
μ green			0.166*** (0.053)
μ foreign			0.102*** (0.054)
Final LL	−8,640.36	−8,400.284	−8,389.202
AIC	17,292.72	16,814.57	16,802.40
BIC	17,335.81	16,864.84	16,888.59
Number of parameters	6	7	12
Observations (*n*)	9,720 (2,430)	9,720 (2,430)	9,720 (2,430)

*Note*: Standard errors in parentheses; the μ parameters are tested against the value of 1, which indicates moderate regret/loss aversion, and a value significantly lower than 1 indicates strong(er) regret/loss aversion. All model comparisons are significant: Comparing RUM and muRRM, the likelihood ratio statistic (LRS) = −2 × (−8,640.4 to −8,400.3) = 480.2 for the model with one regret parameter; this muRRM model consumes one parameter more than the RUM model, implying a critical chi-square value of 3.8 (at a 0.05 level of statistical significance). For the muRRM model with varying regret parameter across choice attributes, the LRS = −2 × (−8,640.4 to −8,389.2) = 502.4; this muRRM model consumes six parameters more than the RUM model, implying a critical chi-square value of 12.59 (at a 0.05 level of statistical significance). For the comparison of the two muRRM models, the LRS = −2 × (−8,400.3 to −8,389.2) = 22.2; the second muRRM model consumes five parameters more than the first muRRM model, implying a critical chi-square value of 11.07 (at a 0.05 level of statistical significance). AIC = Akaike information criterion; BIC= Bayesian information criterion.

*p < .05. **p < .01. ***p < .001 (two-tailed tests).

Based on the standard model in Table 2, we can also calculate marginal rates of substitutions between neighbourhood attributes. These can be calculated by dividing the coefficient value of one choice attribute by the one of another attribute and indicate how much respondents are prepared to sacrifice of one neighbourhood attribute in order to get a more of another attribute. For example, on average, respondents are prepared to accept a 2.88 (95 confidence intervals [CIs]: 2.31–3.60) and 6.18 (95 CIs: 5.21–7.53) percentage point increase in the share of foreigners to obtain a one-minute decrease in the walking time to grocery stores and public transportation, respectively (confidence intervals were calculated using the Krinsky and Robb 1986’s bootstrapping procedure with 2,000 repetitions).

### RRM

When comparing the standard model with different variants of the RRM model, it becomes clear that the latter describes the stated choice behaviour much better than (linear) RUM (see Table 2, also for the likelihood ratio statistics [LRS]). This is indicated by a large difference in model fit – no less than 240 loglikelihood points at the cost of only one additional parameter – as well as by a regret parameter which is close to zero. As elaborated in van Cranenburgh et al. (2015), this latter finding suggests strong regret aversion, that is, a strong asymmetry between regret and rejoice. When we allow the regret parameter to vary across attributes, fit further improves modestly (suggesting a modest variation in terms of regret aversion across attributes). The degree to which the RRM model imposes regret minimization behaviour is determined by the model parameters as well as the occurrence of attribute-level differences in the data (see van Cranenburgh et al. 2015). As a result, we cannot infer the degree of regret minimization behaviour imposed by the model by looking at the estimated parameters only.

**Figure 3.**
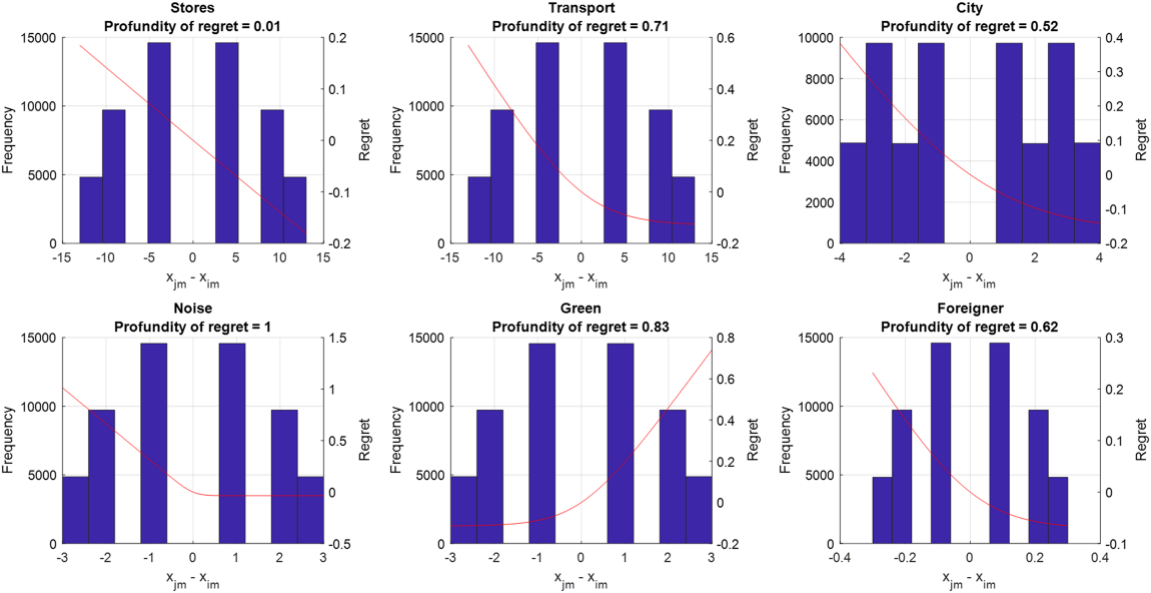
Profundity of Regret.

*Note*: Histograms refer to attribute differences observed in the data.

To accurately assess the degree of regret minimization behaviour, we compute profundity of regret measures *α_m_* and visualize what they imply (see Figure 3). The profundity of regret is a normalized measure (between 0 and 1) that can be computed for each attribute *m*. A high value (*α_m_* > 0.6) indicates a strong degree of regret minimization is being imposed by the estimated model, while a low value (*α_m_* *<* 0.2) indicates a mild degree of regret minimization. It is calculated after estimation of the model using the following equation:

$$\alpha_{m}=\frac{1}{N}\frac{1}{J(J-1)}\sum_{n}\sum_{i}\sum_{\;j\neq i}abs\left[\frac{e^{\left(\beta_{m}/\mu )(x_{\;jmn}-x_{imn}\right)}-1}{e^{\left(\beta_{m}/\mu )(x_{\;jmn}-x_{imn}\right)}+1}\right]$$

Here *N* denotes the total number of choice observations, *J* denotes the number of choice alternatives, *i* and *j* are indices for alternatives, and *x_jmn_* denotes the attribute level of alternative *j* of attribute *m* in choice observation *n*. *β_m_* corresponds the estimated marginal regret of attribute *m*. Readers interested in more details on the profundity of regret measure, its derivation and additional properties are referred to van Cranenburgh et al. (2015).

Figure 3 shows that the estimated RRM model implies the presence of intermediate to strong regret aversion (i.e., *α* > 0.60) for the attributes distance to public transport, noise exposure, share of green areas and share of foreigners. This implies that when comparing neighbourhood alternatives, losses loom larger than equivalently sized gains for most attributes. For example, when the considered alternative has no green areas in the residential area, while a competing alternative has many green areas in the residential area, this causes a considerable amount of regret on the side of the decision-maker (regret, or R ≈ 0.8). However, when the considered alternative has many green areas, while the competing alternative has none, this cause only a moderate amount of rejoice (R ≈ −0.1). Only for the attribute distance to grocery stores we find no regret aversion. Note that as explained in van Cranenburgh et al. (2015), when the regret aversion parameter becomes very close to zero or considerably larger than one, it becomes difficult to identify; in such cases, common practice is to fix the regret parameter's value at either 0.05 (in case of a parameter close to zero) or ten (in case of a parameter considerably larger than one). Regarding the significance levels of the *μ* parameters, for ease of interpretation we test them against the value of 1 which indicates moderate regret/loss aversion and hence a value significantly lower than 1 indicates strong(er) regret/loss aversion.

### Exploring Decision-Rule Heterogeneity, Taste Heterogeneity, and the Potential Relevance of Decision Rules for Macrolevel Outcomes

#### Exploring Heterogeneity in Decision Rules

To test for the presence of decision rule-heterogeneity, we first estimate a two-class latent class choice model where one class is assigned a linear additive utility maximization rule, and the other a regret minimization rule (see model muRRM-RUM in Table 3). For parsimony, we assume one regret aversion parameter in the regret class, which is thus the same for each attribute. The estimated size of the utility maximization-class is 17% of the sample, the other 83% being allocated to the regret minimization class; as explained above, the percentage for the regret class is computed as follows: $\pi_{r}=\frac{\exp(\delta_{\text{r}}=1.6)}{\exp(\delta_{\text{r}}=1.6)+1}$ . In terms of model fit, this model significantly outperforms the best single rule-choice model presented above, highlighting the importance of capturing heterogeneity. Motivated by the small share of utility maximizers in this model, we continue by estimating a latent class model which presumes two regret minimization classes but allows for different degrees of regret aversion (reflected in different regret aversion parameters) across classes (see model muRRM-muRRM in Table 3). The result is a model with a superior model fit compared to the utility-regret mixture. Both classes are of about equal size (51% and 49%), and one class is estimated to have very strong regret aversion, while the other class represents intermediate levels of regret aversion. Note that while it is technically possible to extend the analyses to three or more classes, for the purpose of this study we limit our analyses to a two-class model. Increasing the number of classes would hamper behavioural interpretation (albeit improve statistical performance: the three-class muRRM model attains a BIC of 16,502, against a BIC of 16,608 for the two-class model).

**Table 3.** Latent Class Choice Models Considering Different Decision Rules.

	muRRM-RUM		muRRM-muRRM	
	Class 1 (83%)	Class 2 (17%)	Class 1 (51%)	Class 2 (49%)
*Choice attributes*				
Stores	−0.0248*** (0.002)	−0.0461*** (0.010)	−0.0151*** (0.005)	−0.0329*** (0.005)
Transport	−0.0434*** (0.002)	−0.203*** (0.026)	−0.0309*** (0.005)	−0.084*** (0.005)
City	−0.0925*** (0.009)	−0.563*** (0.063)	−0.00074 (0.037)	−0.256*** (0.037)
Noise	−0.416*** (0.014)	−0.242*** (0.063)	−0.468*** (0.040)	−0.330*** (0.040)
Green	0.358*** (0.017)	0.0937 (0.050)	0.539*** (0.068)	0.140*** (0.068)
Foreign	−1.580*** (0.114)	3.450*** (0.932)	−2.740*** (0.375)	0.343 (0.375)
*Class membership and regret aversion parameters*				
$\delta_{r}$	1.600*** (0.190)		−0.0467 (0.311)	
μ	0.121*** (0.003)		0.0865* (0.003)	0.276*** (0.017)
Final LL	−8,269.95		−8,234.95	
AIC	16,567.90		16,499.90	
BIC	16,595.73		16,529.72	
Number of param.	14		15	
Observations (n)	9,720 (2,430)		9,720 (2,430)	

*Note*: RUM = random utility maximization; RRM = random regret minimization; standard errors in parentheses; the μ parameters are tested against the value of 1, which indicates moderate regret/loss aversion, and a value significantly lower than 1 indicates strong(er) regret/loss aversion. AIC = Akaike information criterion; BIC= Bayesian information criterion.

*p < .05. **p < .01. ***p < .001 (two-tailed tests).

As hinted at earlier, the formulated latent class models cannot distinguish decision-rule heterogeneity from taste heterogeneity, since both taste parameters and decision rules are allowed to vary across classes. While this assumption is behaviourally realistic, one should keep in mind that decision rule-heterogeneity will always be partly confounded with taste heterogeneity. To elaborate, respondents differ amongst each other in terms of their tastes as well as in terms of their employed decision rule. Together, these differences in latent tastes and decision rules result in differences in choice outcomes across respondents. The latent class methodology uses differences in choice outcomes across respondents, to assign one respondent in one class and another respondent in another class. Inevitably, this implies that each class represents a group of respondents whose combined tastes and decision rules are similar within the group yet distinct from that of respondents in the other class. To give a concrete example, it may be noted that the parameter associated with the attribute ‘share of foreigners’ has a different sign in each class (for each of the estimated latent class models); this suggests that in each model, one class captures a dislike of living close to foreigners, while another class captures a preference towards living close to foreigners – this, in addition to the variation in employed decision rules which is also captured in these models. The latent class model can provide an indication regarding decision-rule heterogeneity, but other approaches are better suited if taste heterogeneity is the primary research interest (see below for separate RUM and RRM models on taste heterogeneity).

#### Robustness of Decision Rule-Prevalence, and Heterogeneity in Tastes Across Social Contexts

Oftentimes heterogeneity in tastes of decisions can be associated with observable characteristics. In the context of our data, we can investigate whether heterogeneity is present across the four cities in our sample, that is, to what extent the city context matters for neighbourhood preferences. At the same time, we can examine to what extent loss aversion is present in each city context which indicates how robust the prevalence of the random regret decision rule is. To explore this, we estimate RUM-MNL and muRRM models for each city individually and compare the estimation results. Table 4 shows the results, and the parameter estimates as well as the percentage deviation from the mean for the estimates.^2^

**Table 4.** Random Utility Maximization (RUM) and Random Regret Minimization (RRM) Models per City Context.

	RUM				muRRM			
	Hanover	Mainz	Bern	Zurich	Hanover	Mainz	Bern	Zurich
*Choice attributes*								
Stores	−0.026*** (0.006)	−0.036*** (0.005)	−0.032*** (0.005)	−0.041*** (0.005)	−0.020*** (0.004)	−0.026*** (0.003)	−0.026*** (0.003)	−0.0293*** (0.003)
	[−22%]	[6%]	[−5%]	[21%]	[−22%]	[2%]	[4%]	[16%]
Transport	−0.052*** (0.006)	−0.060*** (0.005)	−0.088*** (0.005)	−0.086*** (0.005)	−0.038*** (0.004)	−0.044*** (0.003)	−0.062*** (0.003)	−0.061*** (0.003)
	[−27%]	[−16%]	[22%]	[20%]	[−26%]	[−15%]	[21%]	[19%]
City	−0.062*** (0.019)	−0.130*** (0.016)	−0.25*** (0.014)	−0.177*** (0.016)	−0.056*** (0.013)	−0.103*** (0.011)	−0.187*** (0.010)	−0.136*** (0.011)
	[−60%]	[−16%]	[62%]	[14%]	[−54%]	[−14%]	[55%]	[13%]
Noise	−0.457*** (0.028)	−0.443*** (0.023)	−0.453*** (0.020)	−0.427*** (0.022)	−0.366*** (0.021)	−0.354*** (0.018)	−0.354*** (0.015)	−0.338*** (0.016)
	[3%]	[0%]	[2%]	[−4%]	[4%]	[0%]	[0%]	[−4%]
Green	0.498*** (0.029)	0.415*** (0.024)	0.402*** (0.021)	0.397*** (0.023)	0.344*** (0.021)	0.29*** (0.017)	0.275*** (0.015)	0.264*** (0.016)
	[16%]	[−3%]	[−6%]	[−7%]	[17%]	[−1%]	[−6%]	[−10%]
Foreign	−2.59*** (0.269)	−1.810*** (0.226)	−0.546*** (0.192)	−0.540*** (0.215)	−1.93*** (0.192)	−1.45*** (0.157)	−0.584*** (0.133)	−0.533*** (0.150)
	[89%]	[32%]	[−60%]	[−61%]	[72%]	[29%]	[−48%]	[−53%]
*Regret aversion parameter*								
μ					0.115** (0.043)	0.142*** (0.031)	0.196*** (0.033)	0.174*** (0.035)
					[−27%]	[−9%]	[25%]	[11%]
Final LL	−1,501.43	−2,047.90	−2,748.66	−2,251.85	−1,449.28	−1,983.09	−2,677.78	−2,195.83
AIC	3,014.86	4,107.81	5,509.33	4,515.70	2,912.56	3,980.19	5,369.57	4,405.66
BIC	3,047.57	4,142.23	5,545.68	4,550.75	2,950.72	4,020.35	5,411.97	4,446.55
*N* of parameters	6	6	6	6	7	7	7	7
Observations (*n*)	1,724 (431)	2,292 (573)	3,160 (790)	2,544 (636)	1,724 (431)	2,292 (573)	3,160 (790)	2,544 (636)

*Note*: Standard errors in (…); percentage deviation from the mean (PDM) for the estimates in […]; for each choice attribute and city *c*, it is calculated by $PDM_{c}=\left(\beta_{c}-\left((1/4)\sum_{c=1}^{4}\;\beta_{c}\right)/(1/4)\sum_{c=1}^{4}\;\beta_{c}\right)\times 100$ ; the μ parameters are tested against the value of 1, which indicates moderate regret/loss aversion, and a value significantly lower than 1 indicates strong(er) regret/loss aversion. AIC = Akaike information criterion; BIC= Bayesian information criterion.

*p < .05. **p < .01. ***p < .001 (two-tailed tests).

Based on Table 4 several observations can be made. Firstly, based on a LRS test we conclude that city specific models are statistically superior over one generic model for all cities (presented in Table 2). This signals that the populations across the four cities are heterogeneous in their preferences. The generic RUM-MNL model attains a final log-likelihood of −8,640.4, while the sum of the final log-likelihoods for the RUM-MNL models per city equals −8,549.8. Therefore, the LRS equals LRS = −2 × (−8,640.4 to −8,549.8) = 181. The city specific models together consume 18 parameters more than the generic model, implying a critical chi-square value of 28.9 (at a 0.05 level of statistical significance). As such, the LRS exceeds the critical chi-square value. The same holds for the city specific muRRM models. Secondly, comparing the model fit between the RUM-MNL and the muRRM across the four cities we observe that for all cities the regret-based models significantly outperform their utility-based counterparts. For each city, the model fit improvement exceeds 50 log-likelihood points. Thirdly, looking at the percentage deviation from the mean for the estimates, we see that the heterogeneity is especially pronounced for the attributes ‘share of foreigner in the residential area’ and ‘distance to city centre’. More specifically, we can see that the residents of the two German cities are relatively more averse to foreigners than their Swiss counterparts, while the residents of the two Swiss cities consider distance to the city centre to be relatively more important than their German counterparts. The differences in preferences for the share of foreigners in the neighbourhood could be explained by existing differences in the share of foreigners and in immigration patterns between the Swiss and German cities. The existing share of foreigners is higher in the Swiss cities (e.g., for 2018, Bern: 24.1; Zurich: 32.3) compared with the German cities (e.g., for 2018, Hanover: 18.7; Mainz: 17.5).^3^ Therefore, on average, the higher exposure to migrants might have a positive effect on their acceptance, following contact theory (even if the type of contact matters and both positive and negative effects are possible, Allport [1954] 1979; Pettigrew and Tropp 2006). Furthermore, while in all cities there is a substantial share of migrants with a non-Western migration background, in the Swiss cities migrants from Germany are a large group and, despite resentments, natives have a more positive attitudes towards this group compared with other migrant groups, especially non-Western (European) migrants (e.g., Diehl et al. 2018). Fourthly, regarding heterogeneity in decision rules, the comparatively larger mu's for the two Swiss cities suggests the Swiss residents behave somewhat less regret/loss averse than their German counterparts. However, considering the difference in the absolute levels, it seems fair to say that overall, the residents are fairly homogenous across cities in terms of the degree to which they act regret/loss averse which supports the robustness of the prevalence of loss aversion across city contexts.

Given the strong indication for heterogeneity across cities, we continue our analyses by estimating a LC model with two muRRM classes, in which the cities enter the class membership function as categorical variables. Table 5 shows the results. In line with the previous results, this model also identifies the heterogeneity across German and Swiss cities. More specifically, residents of the German cities are found to be more likely to choose in accordance with Class 1, while residents of the Swiss cities are more likely to choose in accordance with Class 2. Class 1 is characterized by a strong aversion for the share of foreigners in the residential location, while Class 2 is characterized by a relatively strong preference for residential locations close to the city centre. Furthermore, regarding heterogeneity in decision rules, we see that Class 2 is less regret/loss averse than Class 1. This supports the results presented in Table 4, where we also found that the Swiss residents behave somewhat less regret/loss averse than their German counterparts. The class sizes depend on the city context and vary across cities (Class 1: 74%; Class 2: 26% for Hanover; Class 1: 60%; Class 2: 40% for Mainz; Class 1: 68%; Class 2: 32% for Bern; Class 1: 61%; Class 2: 39% for Zurich).

**Table 5.** Latent Class Choice Model with City Contexts in the Class Membership Function.

	muRRM-muRRM	
	Class 1	Class 2
*Choice attributes*		
Stores	−0.013*** (0.004)	−0.033*** (0.003)
Transport	−0.029*** (0.004)	−0.083*** (0.005)
City	0.014 (0.023)	−0.253*** (0.016)
Noise	−0.456*** (0.026)	−0.345*** (0.017)
Green	0.537*** (0.039)	0.164*** (0.164)
Foreign	−3.060*** (0.308)	0.383 (0.213)
*Class membership and regret aversion parameters*		
μ	0.082* (0.042)	0.270*** (0.038)
γ Hanover	Fixed	−1.070*** (0.268)
γ Mainz	Fixed	−0.392 (0.206)
γ Bern	Fixed	0.770*** (0.233)
γ Zurich	Fixed	0.447*** (0.203)
Final LL	−8,174.24	
AIC	1,6384.48	
BIC	1,6513.76	
Number of param.	18	
Observations (*n*)	9,720 (2,430)	

*Note*: z-Values in parentheses; the μ parameters are tested against the value of 1, which indicates moderate regret/loss aversion, and a value significantly lower than 1 indicates strong(er) regret/loss aversion; AIC = Akaike information criterion; BIC = Bayesian information criterion; RRM = random regret minimization.

*p < .05. **p < .01. ***p < .001 (two-tailed tests).

#### Illustrating Taste Heterogeneity and the Relevance of Decision Rules for Macrolevel Outcomes within a City Context

In the following, based on data for the city of Zurich, we shed more light on taste heterogeneity with regard to the share of foreigners in the neighbourhood and the importance of decision rules for neighbourhood choice probabilities in a specific city context. We rely on the Zurich data as it contains georeferenced information at the respondent level which help to construct a (still hypothetical) city neighbourhood context in line with the choice attributes used in the SCE (more details below).

Since a latent class model comprising different decision rules (as presented in Table 3) captures taste heterogeneity and decision-rule heterogeneity, it is difficult to separate the two types of heterogeneity. To illustrate *taste heterogeneity* beyond the city context (see previous section), in Table 6 we provide separate RUM and muRRM models including interaction effects between the choice attribute on the share of foreigners in the neighbourhood and respondents’ age and migration background (we focus on these determinants as they significantly affect preferences for foreigners, while gender, education and income do not reveal significant effects or are correlated with migration background). The results show for both decision rules an increasing dislike of higher shares of foreigners in the neighbourhood with increasing age, as well as more positive preferences for a higher foreigner share for respondents with a non-Western migration background compared to those with no migration background; there is no significant difference between respondents with a Western migration background and those with no migration background. This is in line with other studies indicating that younger individuals are more tolerant towards ethnic diversity than older ones (e.g., Clark 2009; Farley et al. 1997; Semyonov et al. 2007) and that majority and minority ethnic groups differ in neighbourhood/residential preferences; the majority group expresses stronger in-group preferences than minority groups (e.g., Alba and Logan 1993; Bruch and Swait 2019; Krysan et al. 2009). Yet, the results presented in Table 6 show heterogeneity in in-group preferences across minority groups. While there is no significant difference between those with no migration background and those with Western/European migration background, we observe remarkable differences between no migration background and a non-Western background (Africa/Asia/South America), suggesting that cultural distance might play a role for neighbourhood preferences.

**Table 6.** Random Utility Maximization (RUM) and Random Regret Minimization (RRM), Zurich Data.

	RUM	muRRM
	Zurich	Zurich
*Choice attributes*		
Stores	−0.0414*** (0.005)	−0.0296*** (0.003)
Transport	−0.0861*** (0.005)	−0.0614*** (0.003)
City	−0.178*** (0.017)	−0.137*** (0.011)
Noise	−0.429*** (0.023)	−0.340*** (0.016)
Green	0.396*** (0.023)	0.263*** (0.016)
Foreign	0.941 (0.759)	0.530 (0.512)
*Respondent characteristics interactions between foreign and …*		
Age	−0.038* (0.016)	−0.028* (0.011)
Western migration background	−0.194 (0.681)	−0.041 (0.455)
Non-Western migration background	0.146* (0.591)	1.140* (0.413)
*Regret aversion parameter*		
μ		0.172*** (0.034)
Final LL	−2,240.69	−2,183.769
AIC	4,499.39	4,387.537
BIC	4,561.82	4,445.920
*N* of parameters	9	10
Observations (*n*)	2,536 (634)	2,536 (634)

Note: Standard errors in parentheses; the μ parameters are tested against the value of 1, which indicates moderate regret/loss aversion, and a value significantly lower than 1 indicates strong(er) regret/loss aversion. AIC = Akaike information criterion; BIC = Bayesian information criterion.

*p < .05. **p < .01. ***p < .001 (two-tailed tests).

Apart from preference heterogeneity, the question remains *whether assumptions about decision rules can influence macrolevel outcomes* such as patterns of neighbourhood demand, segregation, and neighbourhood sorting. While we cannot provide an example of actual neighbourhoods at small scale, we use data for the city of Zurich to construct a ‘hypothetical’ city consisting of 12 neighbourhoods where assumptions about neighbourhood choice attribute values (transport, city, noise, etc.) included in the SCE are based on georeferenced survey data for Zurich (see Table 7). We applied the estimated RUM and muRRM models for Zurich (using the estimates as reported in Table 4) to the 12 neighbourhood alternatives to predict the choice probability for each neighbourhood (i.e., the ‘market share’). This shows how the use of a different decision rule can result in a change in an expected neighbourhood demand distribution. Table 7 indicates that, for most neighbourhoods, choice probabilities are, by and large, similar for both decision rules (differences between one and three percentage points), and for two neighbourhoods there are no differences in choice probabilities between RUM and RRM. However, for one neighbourhood we observe a 11 percentage points difference with a 26% probability for RUM and 15% probability for RRM. The high RUM value could be explained by the attractive neighbourhood characteristics such as minimum values for distance to stores and city centre. To see why the RRM model predicts a much lower market share is not immediately apparent. Inspection of the regret computations shows that the first neighbourhood (neighbourhood identification, NID = 1) incurs most regret from the choice attribute green areas. This attribute is both relatively important and the first neighbourhood performs poor on this attribute relative to other neighbourhoods. Even if the differences are not large across all neighbourhoods, the example in Table 7 illustrates that decision rules potentially matter for macrolevel outcomes.

**Table 7.** Neighborhood Characteristics, RUM and muRRM Choice Probabilities, Hypothetical City Example Based on Zurich Data.

	Choice attributes						Choice probabilities		
NID	Stores	Trans-port	City	Noise	Green	Foreign	RUM	muRRM	Diff.
1	2	5	1	1	2	22.22	0.26	0.15	0.11
2	5	10	4	3	4	15.72	0.10	0.12	−0.01
3	10	15	3	3	3	23.45	0.04	0.06	−0.02
4	5	5	2	4	1	27.34	0.03	0.03	0.00
5	10	2	2	4	1	18.06	0.04	0.03	0.00
6	10	10	3	3	3	15.88	0.07	0.10	−0.03
7	10	15	4	1	4	16.00	0.14	0.11	0.03
8	5	15	3	3	3	17.98	0.05	0.08	−0.03
9	5	10	4	2	3	30.77	0.09	0.11	−0.02
10	15	10	4	3	4	24.77	0.06	0.08	−0.02
11	15	15	4	3	3	32.95	0.03	0.04	−0.01
12	5	10	4	3	4	35.61	0.08	0.09	0.01

*Note*: The 12 neighbourhoods (NID 1–12) were constructed based on data for 12 large districts in the City of Zurich. For each neighbourhood, we assigned attribute values in line with the stated choice experiment (stores and transport in walking minutes; distance to city centre in km, noise from 1 (*none*) to 4 (*high*), green areas from 1 (*none*) to 4 (*many*), share of foreigner in %. For each respondent in our data, we have georeferenced data regarding distance to nearest public transport options and stores, as well as road traffic noise in db at the house/flat, share of green space in 100 m surrounding; the information was averaged across respondents per neighbourhood and categorized in line with the stated choice experiment. We used Google Maps to convert average distances in km to walking minutes where needed. The foreign attribute value is based on the observed share of non-Western migrants per neighbourhood in our data. RRM models are choice set composition dependent models. Therefore, the estimates or RRM models pertain to a given choice set size. In case an RRM model is applied to predict demand in a choice set with a different size, compared to what it was estimated on, a choice set size correction factor must be used (2/J) (see van Cranenburgh et al. 2015 for details). AIC = Akaike information criterion; BIC = Bayesian information criterion; RRM = random regret minimization; RUM = random utility maximization.

## Discussion and Conclusions

Analysing individual decision-making can inform sociological research regarding the explanation of individual behaviour as part of microfoundations and micro–macro explanations of social phenomena. It also helps to understand better how individual behaviour depends on and varies across social contexts. While there has been much progress in decision-making research in the last decades, this has hardly been acknowledged in sociology. On the other hand, integrative theories of action in sociology (e.g., Esser and Kroneberg 2015; Lindenberg 2008; Vaisey 2009) are built on or are in line with insights of dual process theories, comprising different modes of action – automatic-spontaneous and reflecting-calculating modes (Kahneman 2011). This is an important step towards an explanation of individual action, which is more realistic and has higher explanatory power than simpler behavioural models. The two decision rules considered in this article belong to the reflecting-calculating mode of action and, as such, their relevance and context-dependence can be studied in dual process research.

Notwithstanding these recent developments, parsimonious theories assuming utility maximizing actors are still popular in sociological and other social science research. Our study combines insights from choice modelling and SCE research to demonstrate how heterogeneity in decision-making can be examined. To what extent can individuals in a population be described by utility maximization or loss aversion? Often, researchers assume some kind of behavioural model such as maximizing behaviour without testing its underlying assumptions and whether there is a (dis-)connect between theory and statistical model. Here, discrete choice theory and choice modelling techniques provide a much closer link between theoretical foundations and statistical analysis. Together with empirical methods of data collection such as SCEs, choice modelling offers an environment where different decision rules and hence assumptions about individual behaviour, as well as their context dependence, can be studied in population surveys as well as revealed preferences and process-generated data (Chorus 2014).

This advances sociological research because it allows to test different decision rules and to find out what behavioural assumptions seem more realistic. Across all analyses of our data, we find that regret minimization describes the stated choice behaviour much better than a linear in parameters utility maximization model, suggesting decision-makers are best described as loss averse (similar to Tversky and Kahneman 1991): comparing attributes of neighbourhood alternatives, ‘losses’ in neighbourhood attributes such as environmental conditions loom larger than gains of equal size. In fact, our study revealed that very strong regret aversion exists for the three neighbourhood attributes: the share of green areas, the share of foreigners, and exposure to noise. The approach presented in this study does not only allow to differentiate between different decision rules but also, based on a SCE design, to examine the importance of decision rules at the level of choice attributes. In this regard, the relevance of loss aversion for the ethnic composition of a neighbourhood as well as environmental (noise) pollution might be informative for (political) intervention strategies. For example, it can be considered in framing environmental and community programs. On the other hand, it might be questioned whether the RUM and RRM assumptions matter at all for societal outcome (similar to the influence of assumptions about educational decision-making on educational inequalities, e.g., Barone et al. 2021: 149). In other words, although our approach can show that one decision rule describes decision-making better than the other, this might have no relevance for macrolevel outcomes such as neighbourhood demand and sorting. While this might depend on the specific case at hand, using a hypothetical neighbourhood example informed by our data we illustrated that RUM and RRM assumptions can make a difference for macrolevel outcomes such as the expected demand for neighbourhoods (this has also been demonstrated for mobility networks and aggregate mobility forecasts in the transport sector, see Bekhor et al. 2012; van Cranenburgh and Chorus 2018).

Our findings are broadly in line with research documenting the relevance of loss aversion in decision-making (Mrkva et al. 2019). The RRM model gained popularity in choice modelling research, and our empirical application demonstrates its usefulness for sociological research. It seems therefore desirable to test it against the standard model of RUM and the oftentimes (implicitly) employed assumption of utility maximization in sociological research. While SCE data has advantages regarding the separation of attribute effects in decision-making, it needs to be stressed that the RUM and RRM model can also be tested with revealed preference data (see, e.g., van Cranenburgh and Chorus 2018). We do not suggest that RRM always outperforms RUM, but we call for considering competing behavioural assumptions more often in decision-making research in sociology. This will allow a more direct testing of sociological theories relying on loss aversion, and of the relevance of loss aversion across different social contexts; some social contexts might be prone to more (or less) loss aversion (or utility maximizing behaviour). We are aware that there are many possible decision rules, and various rules should be tested; however, loss aversion is a good starting point because it is a universal phenomenon (Camerer 2004, 2005; Heath et al. 1999; Mrkva et al. 2019). Also, the RRM model has been implemented in many software packages and hence can be easily applied by researchers. Examples include the following:

- the Apollo package for R (Hess and Palma 2019),
- the randregret command for Stata (Gutiérrez-Vargas et al. 2021),
- the RRM model is also included in NLogit (https://www.limdep.com/products/nlogit/),
- in LatentGOLD (https://www.statisticalinnovations.com/latent-gold-6-0/) and
- Biogeme (https://biogeme.epfl.ch/) (for more options see https://www.advancedrrmmodels.com/).

Our study can be seen as a starting point for bridging choice modelling and sociological research regarding the study of decision rules and decision rule-heterogeneity. Our empirical application is limited by the fact that neighbourhood choice is a (much) more complex process than presented in our SCE. For example, it typically includes multistage decision-making (e.g., Bruch and Swait 2019 for modelling such processes) as well as joint decision-making in households (e.g., Timmermans et al. 1992 for a choice modelling approach). Also, there might be more attributes such as access to schools that are relevant for this process (at least for subgroups in the population), but which were not included in our experiment. On the other hand, our experiment clearly shows the potential for this type of choice modelling research for sociology. We were able to uncover the relevance of RRM and hence loss aversion and in principle this can also be considered in multi-stage choice models as well as modelling joint decision-making; for example, household members can differ in their decision-making processes.

Future research can build upon this study and address important issues such as the following: First, in the light of dual process theories (Kahneman 2011; Kroneberg 2014; Vaisey 2009), it can be studied to what extent individuals employ decision heuristics such as elimination-by-aspect or reflecting-calculating rules such as utility maximization and loss aversion and under what conditions (or social contexts) each of the different rules is more likely. RRM and loss aversion refer to reflective-calculating behaviour and bringing in automatic-spontaneous decision rules will make the analysis not only more realistic but also allows studying dual processes in decision-making. Second, more generally it is important to better understand what affects the use of different decision rules, for example how attitudes affect decision-making in this regard. Third, the relative importance of taste heterogeneity and decision-rule heterogeneity needs more attention. Our latent class analysis suggests that the majority of individuals follow regret minimization whereas the minority follows utility maximization. While often it seems unrealistic that all individuals can be best described by a single behavioural model, the latent class modelling approach captures both taste and decision-rule heterogeneity and this makes it difficult to exactly separate the importance of taste heterogeneity and decision-rule heterogeneity. The prevalence of heterogeneity in decision rules can be further explored by using other approaches such as artificial neural networks (see van Cranenburgh and Alwosheel 2019). Fourth, in the light of sociological research interests decision rule-heterogeneity can be combined with investigating the importance of social contexts for decision-making, for example by combining framing and SCEs (e.g., Carlsson et al. 2010 for social norms and ethical consumption), social interactions, for example by modifying choice models and social network models (e.g., Pink et al. 2020; Zeng and Xie 2008), as well as joint decision-making, for example, by investigating household decision-making and corresponding behavioural rules (e.g., Timmermans et al. 1992). Data on decision rule-heterogeneity can also inform agent-based models (Bruch and Atwell 2015) and this way help to shed more light on micro–macro links in sociological research. Informed by our stated preference data and decision rule models we could illustrate that the decision rule can affect macrolevel outcomes such as neighbourhood demand. Such micro–macro links can be studied more in-depth in future research.

While we studied decision rules, decision rule-heterogeneity with an application to neighbourhood choice, the approach presented in this article and its extensions can provide valuable insights in many fields of sociological research including educational decision-making, migration, friendship choices and deviant behaviour. In conclusion, we would like to echo the observation by Bruch and Feinberg (2017) that, albeit sociological phenomena are complex and choice modelling approaches cannot often be readily applied to these phenomena, there is great potential where both fields – choice modelling and sociology – can complement each other.

## Supplemental Material

sj-pdf-1-smr-10.1177_00491241231186657 - Supplemental material for Maximizing Utility or Avoiding Losses? Uncovering Decision Rule-Heterogeneity in Sociological Research with an Application to Neighbourhood Choice Supplemental material, sj-pdf-1-smr-10.1177_00491241231186657 for Maximizing Utility or Avoiding Losses? Uncovering Decision Rule-Heterogeneity in Sociological Research with an Application to Neighbourhood Choice by Ulf Liebe, Sander van Cranenburgh, and Caspar Chorus in Sociological Methods & Research

## Notes

1. While we did not preregister this survey study and stated choice experiment, we can confirm that the data were not used to generate and test hypotheses at the same time. It was the aim of the stated choice experiment to explore the importance of neighborhood attributes for neighborhood choice as well as the relevance of RUM and RRM decision rules.

2. See also Table A2 in the Online Appendix for random parameter logit models based on the pooled data and data per city, indicating robustness of the main findings when assuming that effects of single attributes can vary in the population.

3. Information on the share of foreigner per city can be obtained from official sources (Hanover: https://www.hannover.de/Leben-in-der-Region-Hannover/Verwaltungen-Kommunen/Die-Verwaltung-der-Landeshauptstadt-Hannover/Dezernate-und-Fachbereiche-der-LHH/Stadtentwicklung-und-Bauen/Fachbereich-Planen-und-Stadtentwicklung/Stadtentwicklung/Bev%C3%B6lkerungsentwicklung-und-demografischer-Wandel; Mainz: https://www.edoweb-rlp.de/resource/edoweb:7037097/data; Bern: https://www.bern.ch/politik-und-verwaltung/stadtverwaltung/bss/fachstelle-fuer-migrations-und-rassismusfragen/fakten; Zurich: https://www.stadt-zuerich.ch/prd/de/index/statistik/themen/bevoelkerung/nationalitaet-einbuergerung-sprache/anteil-auslaendische-bevoelkerung.html).
