# Supplementary material for: Maximizing Utility or Avoiding Losses? Uncovering Decision Rule-Heterogeneity in Sociological Research with an Application to Neighbourhood Choice
Source: Sociol Methods Res. 2023 Jul 18;54(1):275–314. doi: 10.1177/00491241231186657 (PMC11788871; doi:10.1177/00491241231186657)
Supplement: sj-pdf-1-smr-10.1177_00491241231186657 - Supplemental material for Maximizing Utility or Avoiding Losses? Uncovering Decision Rule-Heterogeneity in Sociological Research with an Application to Neighbourhood Choice [file sj-pdf-1-smr-10.1177_00491241231186657.pdf]

Appendix

Table A1. Correlations Between Choice Experiment Attributes and Respondent Characteristics (Experimental Balance Check).

Variables	(1)	(2)	(3)	(4)	(5)	(6)
Choice attributes						
(1) Stores	1.000					
(2) Transport	0.005	1.000				
(3) City	0.006	−0.007	1.000			
(4) Noise	−0.011	−0.003	−0.007	1.000		
(5) Green	−0.004	−0.004	−0.000	−0.002	1.000	
(6) Foreign	0.003	−0.004	0.004	0.002	−0.004	1.000
Respondent characteristics						
(7) Woman	0.002	0.004	0.003	0.001	0.002	−0.003
(8) Age	−0.002	−0.001	−0.000	0.000	−0.002	−0.003
(9) Education	−0.007	−0.003	0.001	0.007	−0.006	0.005
(10) Income	−0.008	−0.002	0.004	0.007	−0.005	0.002
(11) Western migration background	0.004	0.001	0.002	0.001	−0.002	−0.001
(12) Non-Western migration background	−0.007	−0.002	0.003	0.000	0.007	0.005
(13) Mainz	0.001	0.002	−0.001	0.001	−0.001	0.001
(14) Hannover	−0.003	0.002	−0.003	0.002	−0.001	−0.008
(15) Bern	0.007	−0.002	0.001	−0.006	0.003	0.007
(16) Zurich	−0.005	−0.002	0.002	0.003	−0.001	−0.001

Table A2. Random Parameter Logit Models, RUM and RRM, Pooled and per City Sample.

	Pooled		Hanover		Mainz		Bern		Zurich	
	RUM	muRRM	RUM	muRRM	RUM	muRRM	RUM	muRRM	RUM	muRRM
Choice attributes										
Stores	−0.0386***	−0.0296***	−0.0386***	−0.028***	−0.0315***	−0.035***	−0.0360***	−0.034***	−0.0473***	−0.037***
	(0.003)	(0.002)	(0.006)	(0.007)	(0.008)	(0.005)	(0.006)	(0.005)	(0.006)	(0.005)
Transport	−0.0927***	−0.0767***	−0.0690***	−0.060***	−0.0664***	−0.064***	−0.116***	−0.106***	−0.108***	−0.085***
	(0.003)	(0.0032)	(0.006)	(0.007)	(0.008)	(0.006)	(0.008)	(0.008)	(0.008)	(0.006)
City	−0.208***	−0.171***	−0.149***	−0.080***	−0.0823***	−0.134***	−0.322***	−0.290***	−0.221***	−0.186***
	(0.012)	(0.009)	(0.022)	(0.021)	(0.025)	(0.019)	(0.023)	(0.022)	(0.023)	(0.017)
Noise	−0.571***	−0.490***	−0.540***	−0.586***	−0.597***	−0.516***	−0.602***	−0.564***	−0.559***	−0.457***
	(0.019)	(0.016)	(0.032)	(0.053)	(0.048)	(0.035)	(0.036)	(0.038)	(0.037)	(0.0289)
Green	0.526***	0.407***	0.483***	0.587***	0.625***	0.441***	0.534***	0.463***	0.506***	0.379***
	(0.019)	(0.016)	(0.032)	(0.058)	(0.050)	(0.036)	(0.034)	(0.037)	(0.037)	(0.029)
Foreigner	−1.495***	−1.27***	−2.182***	−2.94***	−3.234***	−2.17***	−0.627*	−0.740***	−0.729**	−0.679***
	(0.142)	(0.112)	(0.297)	(0.369)	(0.376)	(0.285)	(0.248)	(0.217)	(0.261)	(0.201)
Standard deviation										
Stores	0.0385***	0.019***	−0.000197	0.054***	0.0553***	−0.0247*	0.0481**	0.0368***	0.0444**	0.028*
	(0.009)	(0.007)	(0.022)	(0.013)	(0.017)	(0.011)	(0.015)	(0.013)	(0.017)	(0.012)
Transport	0.0696***	−0.067***	0.0381*	0.053***	0.0471**	0.0467***	0.0961***	−0.0943***	0.0706***	−0.058***
	(0.006)	(0.005)	(0.016)	(0.014)	(0.018)	(0.012)	(0.011)	(0.010)	(0.013)	(0.010)
City	0.299***	−0.231***	0.279***	−0.183***	0.236***	0.260***	0.311***	0.276***	0.302***	−0.222***
	(0.018)	(0.016)	(0.035)	(0.041)	(0.047)	(0.033)	(0.034)	(0.031)	(0.035)	(0.029)
Noise	−0.183***	−0.245***	0.00675	0.289***	0.146	−0.195***	0.301***	0.350***	−0.176*	−0.236***
	(0.040)	(0.024)	(0.200)	(0.063)	(0.119)	(0.051)	(0.055)	(0.046)	(0.087)	(0.049)
Green	0.289***	0.297***	0.151	−0.421***	0.333***	−0.282***	0.266***	−0.348***	0.377***	0.312***
	(0.030)	(0.023)	(0.090)	(0.064)	(0.068)	(0.044)	(0.060)	(0.046)	(0.053)	(0.040)

(continued)

Table A2. Continued

	Pooled RUM	muRRM	Hanover RUM	muRRM	Mainz RUM	muRRM	Bern RUM	muRRM	Zurich RUM	muRRM
Foreigner	−2.613***	2.43***	−3.283***	−3.51***	2.870***	3.87***	−2.217***	−2.81***	1.428	−1.89***
	(0.142)	(0.190)	(0.509)	(0.563)	(0.699)	(0.435)	(0.631)	(0.435)	(0.947)	(0.454)
Regret aversion parameter										
μ		0.220***		0.222***		0.199***		0.287***		0.252
		(0.029)		(0.076)		(0.053)		(0.063)		(0.059)
Final LL	−8,531.129	−8,225.108	−1,487.646	−1,414.689	−2,022.314	−1,927.537	−2,699.310	−2,590.099	−2,220.750	−2,149.279
AIC	17,086.258	16,476.216	2,999.292	2,855.379	4,068.628	3,881.073	5,422.62	5,206.197	4,465.5	4,324.558
BIC	17,172.441	16,569.581	3,064.721	2,926.260	4,137.474	3,955.657	5,495.3199	5,284.955	4,535.5979	4,400.497
Number of parameters	12	13	12	13	12	13	12	13	12	13
Observation (n)	9,720 (2,430)	9,720 (2,430)	1,724 (431)	1,724 (431)	2,292 (573)	2,292 (573)	3,160 (790)	3,160 (790)	2,544 (636)	2,544 (636)

Note: A normal distribution is assumed for all attributes; standard errors in parentheses, all models based on 1,000 Halton draws. AIC = Akaike information criterion; BIC = Bayesian information criterion; RRM = random regret minimization; RUM = random utility maximization.
*$p < .05$. **$p < .01$. ***$p < .001$ (two-tailed tests).
